# Circulating prostaglandin E_2_ concentrations decrease at birth in premature lambs

**DOI:** 10.3389/fped.2025.1636459

**Published:** 2025-11-28

**Authors:** Timothy J. R. Panneflek, Janneke Dekker, Kelly J. Crossley, Cailin Diedericks, Kristel L. A. M. Kuypers, Ebony R. Cannata, Paige J. Riddington, Femmie E. Bloem, Alison M. Thiel, Thomas van den Akker, Graeme R. Polglase, Arjan B. te Pas, Stuart B. Hooper, Indya M. Davies

**Affiliations:** 1 Division of Neonatology, Department of Paediatrics, Willem-Alexander Children’s Hospital, Leiden University Medical Centre, Leiden, Netherlands; 2 The Ritchie Centre, Hudson Institute of Medical Research, Clayton, VIC, Australia; 3 Department of Obstetrics and Gynaecology, Monash University, Clayton, VIC, Australia; 4 Department of Obstetrics and Gynaecology, Leiden University Medical Center, Leiden, Netherlands; 5 Athena Institute, VU University, Amsterdam, Netherlands; 6 Department of Paediatrics, Monash University, Clayton, VIC, Australia

**Keywords:** prostaglandins, perinatal transition, preclinical, ventilation, cord clamping

## Abstract

**Rationale:**

During pregnancy, prostaglandin E_2_ (PGE_2_) is released from the placenta and circulates in relatively high concentrations in the fetus. As PGE_2_ suppresses breathing, PGE_2_ concentrations must decrease after birth, but the timing and mechanisms behind this decrease are unknown. We hypothesised that both umbilical cord clamping and lung aeration contribute to the reduction in PGE_2_ concentrations after birth.

**Materials and methods:**

Instrumented premature lambs (138–141 days gestation) were randomised to receive either physiological-based cord clamping (PBCC; cord clamping after ventilation onset; *n* = 5) or immediate cord clamping (ICC; before ventilation onset; *n* = 6). PGE_2_ concentrations were measured in pulmonary and carotid arterial blood 30 s after ventilation onset, after lung aeration and 30 s after cord clamping. All PGE_2_ data are expressed relative to fetal PGE_2_ concentrations.

**Results:**

Relative to fetal concentrations, ventilation onset decreased PGE_2_ concentrations in the carotid (*p* = 0.036) and pulmonary arteries (*p* = 0.052) in PBCC lambs, whereas cord clamping had no further additional effect on PGE2 concentrations in these lambs. In ICC lambs, cord clamping decreased PGE_2_ concentrations, relative to fetal concentrations, in both the carotid (*p* = 0.001) and pulmonary arteries (*p* < 0.001). Ventilation onset further decreased PGE_2_ concentrations in both the carotid (*p* = 0.002) and pulmonary arteries (*p* = 0.014).

**Conclusion:**

Both umbilical cord clamping and ventilation onset independently decrease PGE_2_ concentrations immediately after birth, which may enhance breathing activity, although the effect of cord clamping is reduced by ventilation onset.

## Introduction

Lung aeration at birth triggers the cardiopulmonary changes that characterise the transition of a fetus into a neonate and is largely achieved by hydrostatic pressure gradients generated by spontaneous breathing or positive pressure inflations (1). Lung aeration not only initiates the onset of pulmonary gas exchange, but also stimulates a large decrease in pulmonary vascular resistance (2). The resulting large increase in pulmonary blood flow (PBF) plays a vital role in sustaining cardiac output after birth by taking over the role of supplying preload for the left ventricle following umbilical cord clamping (hereafter cord clamping) (3).

Although non-invasive respiratory support is now the preferred approach for supporting premature infants at birth (4), recent studies in both animals and humans have shown that the success of this approach is dependent upon the presence of spontaneous breathing (5, 6). This is because the larynx actively adducts during apnoea, thereby sealing the airways and obstructing non-invasive respiratory support and only opens during spontaneous breathing (5, 6). Thus, the success of non-invasive ventilation after birth relies heavily on stimulating spontaneous breathing, while avoiding factors that may inhibit breathing. These factors involve transient hypoxia, antenatal inflammation, the infant's arousal state and circulating mediators that can inhibit breathing (4), which include prostaglandin E_2_ (PGE_2_) and adenosine (7, 8). Both hypoxia and inflammation increase production of prostaglandins (PGs), particularly PGE_2_ (8–11), which inhibits breathing by direct action on the brainstem. PGE_2_ is thought to bind to receptors and alter the excitability of nerve cells responsible for the central pattern generator for breathing by hyperpolarising cell membranes and thereby lowering neuronal firing rates (12). Indeed, PGE_2_ is thought to lower phrenic nerve output, as inhibiting PGE_2_ production with indomethacin increases peak phrenic nerve activity in newborn piglets (13).

*In utero*, PGE_2_ is produced and released by the placenta, circulates in the fetus in relatively high concentrations, and plays a role in the episodic nature of fetal breathing movements (FBMs) (14, 15). Hours-to-days after birth, circulating PGE_2_ concentrations decrease significantly and may contribute to the onset of continuous spontaneous breathing in the newborn (16–18). However, the relationship between breathing activity and PGE_2_ concentrations is complex, as FBMs occur *in utero* and continuous breathing can commence after birth even when PGE_2_ concentrations are high (19). While the decrease in circulating PGE_2_ appears to be associated with cord clamping, which removes access to the main source of PGE_2_ production (i.e., the placenta) (20), the increase in PBF associated with lung aeration may also explain this decrease (21). The lung is the primary site of prostaglandin metabolism after birth, because it contains a high expression of 15-hydroxyprostaglandin dehydrogenase (15-PGDH) (22), the rate-limiting step for prostaglandin metabolism, which converts PGE_2_ into the stable prostaglandin E metabolite (PGEM) (23). Therefore, the redirection of right ventricular output through the lungs, following a decrease in pulmonary vascular resistance, would be expected to markedly increase prostaglandin metabolism and reduce circulating PGE_2_ concentrations (2, 16). However, as circulating PGE_2_ concentrations must reflect a balance between production vs. metabolism, the factors regulating circulating PGE_2_ concentrations after birth are unclear (16, 17, 24). Accordingly, the objective of this study was to investigate how circulating PGE_2_ concentrations change in response to cord clamping and lung aeration (either before or after cord clamping) in the immediate newborn period.

## Material and methods

### Ethical approval

All animal procedures were approved by the Monash Medical Centre Animal Ethics Committee (MMCA 2019/30) and were conducted as stipulated by the National Health and Medical Research Council code of practice for the care and use of animals for scientific purposes (25). Methodological reporting is per the relevant ARRIVE guidelines (26).

### Experimental protocol

Blood samples described in this study were collected from premature lambs included in a separate study (27), with all samples being collected 30 min before the onset of that study. This was designed to minimise the number of animals subjected to experimental procedures. As such, methods for the animal preparation have been described previously (27) and will be only described briefly here.

Twin pregnant ewes (138–141 days of gestation) were initially anaesthetised (Pentothal, i.v. 20 mg/kg: Jurox, New South Wales, Australia) and intubated, before anaesthesia was maintained with inhaled isoflurane (Isoflow, 1.5%–2.5%, Abbott Pty. Ltd., New South Wales, Australia) in air/oxygen mixture (30% oxygen). Ewes were monitored regularly (heart rate, oxygenation, expired CO_2_ and absent corneal reflex) to ensure adequate anaesthesia and maternal wellbeing. Each fetus was instrumented with catheters (internal diameter: 0.86 mm, external diameter: 1.52 mm, Dural Plastic Inc., New South Wales, Australia) in the right carotid and left pulmonary arteries (blood samples) and right jugular vein (anaesthesia drug administration). Transonic blood flow probes were placed around the left carotid (3 mm, Transonic System, Ithaca, NY) and pulmonary (4 mm, Transonic System, Ithaca, NY) arteries. Lambs were intubated (4 mm cuffed endotracheal tube) and their body temperatures were measured using a rectal temperature probe to correct their arterial blood gas measurements. All physiological measurements and ventilation parameters were digitally recorded using a data acquisition system running Labchart v8 software (Powerlab; ADInstruments, New South Wales, Australia).

### Delivery and ventilation protocol

Following delivery, lambs were sedated (Alfaxane 10 mg/mL, 5 mL/h; Jurox New Zealand Pty. Ltd., New Zealand). Anaesthesia in the lamb was maintained using alfaloxone diluted in 5% glucose (Alfaxan, 5–15 mL/kg/h; Jurox New Zealand Pty. Ltd., New Zealand). Lambs were subsequently randomised to receive either physiological-based cord clamping (PBCC; *n* = 5) or immediate cord clamping (ICC; *n* = 6) to separate the effects of cord clamping and ventilation onset on circulating PGE_2_ concentrations. PBCC lambs were mechanically ventilated using air until their lungs were aerated before the umbilical cord was clamped. Lung aeration was defined by an increase in PBF that resulted in an absence of retrograde flow (i.e., blood flow out of the lungs) during diastole (3–5 min after ventilation onset). Lambs randomised to ICC (ICC lambs) had their cords clamped prior to the onset of ventilation.

Intermittent positive pressure ventilation was set in volume-guaranteed mode with a tidal volume of 7 mL/kg estimated body weight (Babylog 8000 Plus, Dräger, Germany), a respiratory rate of 60 breaths/min (0.4/0.6 s, Ti/Te), maximum peak inflation pressures of 35 cmH_2_O, a positive end-expiratory pressure of 5 cmH_2_O and, following cord clamping, oxygen was given as needed based on the lamb's oxygen levels.

### Blood sample protocol

Blood samples of 1 mL were collected from the pulmonary artery, carotid artery and umbilical vein for blood gas analyses and measurements of PGE_2_ concentrations; indomethacin was added to the blood collection tubes to prevent post-collection prostaglandin synthesis. In PBCC lambs, blood samples were collected while the fetus was *in utero*, 30 s after ventilation onset, directly after lung aeration (indicated by the increase in PBF and absence of retrograde flow) and 30 s after cord clamping. In ICC lambs, blood samples were collected while the fetus was *in utero*, 30 s after cord clamping, 30 s after ventilation onset and after lung aeration. A protocol amendment was made halfway through the experiment to also collect a blood sample from the umbilical vein after cord clamping. This was performed in 6 lambs.

### Postmortem protocol

Ewes were euthanised with an intravenous sodium pentobarbitone solution (>100 mg/kg; Lethabarb, Virbac Pty. Ltd., New South Wales, Australia) after both twins were delivered. Newborn lambs were also euthanised with intravenous sodium pentobarbitone solution (>100 mg/kg) following experimental procedures. In addition, postmortem analyses were performed to record body and organ weights. The right lung of each lamb was fixed and analysed to demonstrate the presence of 15-PGDH.

### Physiological recording analysis

PBF and carotid artery blood flow were continuously measured and recorded using LabChart throughout the delivery and ventilation periods.

### PGE_2_ concentration analysis

The blood samples were analysed to determine the primary outcome of circulating PGE_2_ concentrations (pg/mL) using a commercially available bovine monoclonal PGE_2_ ELISA kit (cat# 514010, Cayman Chemicals, Ann Arbor, MI, United States). The analysis was performed according to the manufacturer's instructions, with the methodology described in Supplementary File S1.

### Prostaglanin E metabolite (PGEM) concentration analysis

Circulating concentrations of PGEM were measured using a commercially available monoclonal ELISA kit (cat# 514531, Cayman Chemicals, Ann Arbor, MI, United States), according to manufacturer's instructions (described in Supplementary File S1).

### 15-hydroxyprostaglandin dehydrogenase (15-PGDH) analysis

At postmortem examination, the right lungs of all lambs were pressure fixed (20 cmH_2_O) with formalin. Following fixation, the lungs were cut into 5 mm transverse sections, before the slices were further subdivided with different lung sections selected at random. The randomly selected lung tissue was paraffin-embedded and stained for the presence of 15-PGDH; as specified in Supplementary File S1. The 15-PGDH analysis was displayed in Supplementary File S2 (Figure S3).

### Statistical analysis

All continuous data were presented as mean ± standard error of the mean (SEM). Binary data were presented as *n* (%). Missing data were noted in the results section. All PGE_2_ and PGEM data were described and graphically displayed in Supplementary File S2 (Table S1, Figure S2). PGE_2_ and PGEM concentrations in the fetal umbilical vein were compared with concentrations in the carotid artery and pulmonary artery using a Paired-Samples T-test in 6 lambs. As basal fetal PGE_2_ concentrations were quite variable between lambs [Supplementary File S2 (Figure S3)], carotid and pulmonary artery PGE_2_ concentrations were expressed as a percentage of fetal PGE_2_ concentrations measured in each lamb. This ensured that the changes associated with ventilation and cord clamping were not obscured by the large variability in basal values between lambs. The PGE_2_ data were then transformed (square root) and analysed over time with a One-Way Repeated Measures Analysis of Variance (ANOVA). Differences at each time point within each group (PBCC and ICC) were analysed using *post-hoc* Fisher's least significant differences tests. The results of the *post-hoc* tests are depicted in the text results and Figure 3. In addition, the change in PGE_2_ concentrations in response to ventilation (combined ventilation onset and lung aeration samples) and cord clamping were also separately analysed with Paired-Samples *t*-tests for both PBCC and ICC lambs. This analysis was performed to evaluate the specific effects of cord clamping and ventilation of the lung on PGE_2_ concentrations, irrespective of whether cord clamping or ventilation occurred first. PGEM concentrations were analysed over time with a One-Way Repeated Measures Analysis of Variance (ANOVA). Differences between specific treatments within each group were also analysed with *post-hoc* Fisher's least significant differences tests. The outcomes of the PGEM analyses were reported in the text results. The researchers could not be blinded during sample collection and analysis due to the nature of the study.

**Figure 1:**
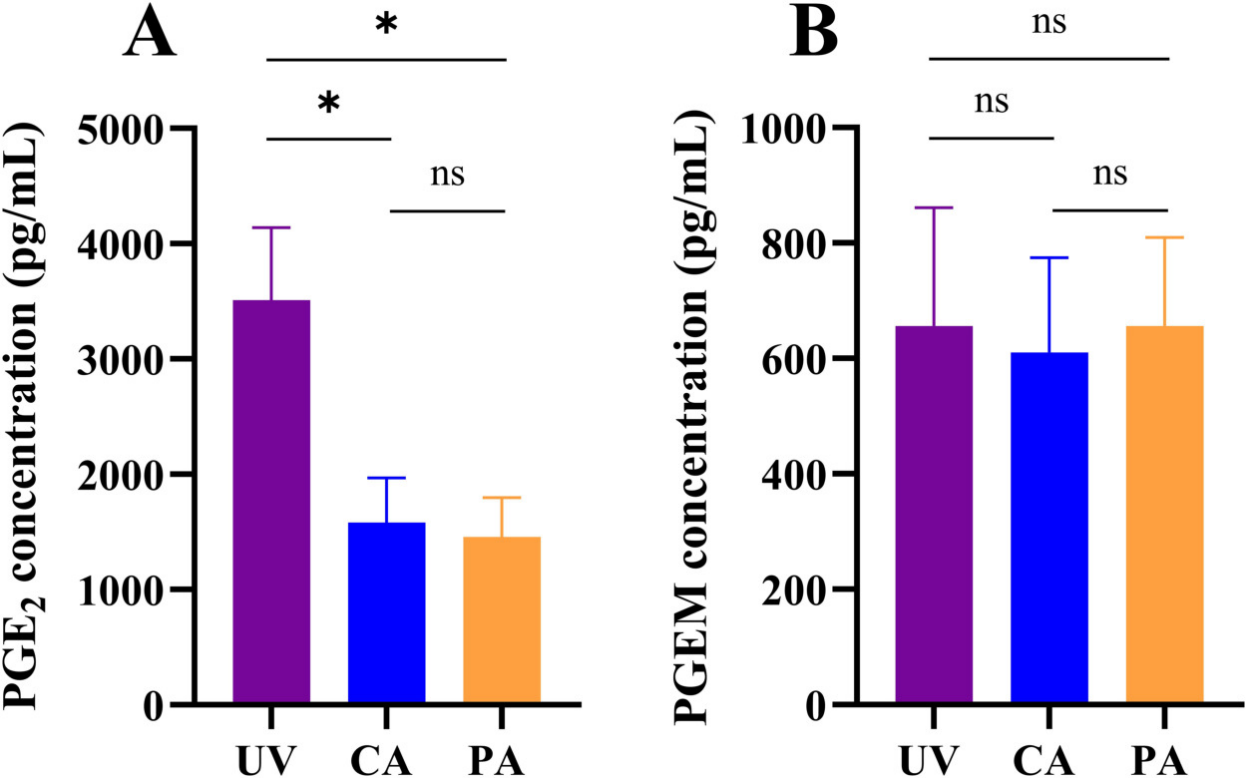
Prostaglandin E_2_ (PGE_2_) and prostaglandin E metabolite (PGEM) concentrations in the fetal circulation. PGE_2_ **(A)** and PGEM **(B)** concentrations measured in the umbilical vein (UV), carotid artery (CA) and pulmonary artery (PA) in *n* = 6 fetal sheep. Data were presented as mean ± standard error of the mean. * represents a *p*-value ≤0.05; ns represents a *p*-value >0.05.

Statistical analyses were performed with IBM SPSS Statistics V.29.0 (IBM Software, Chicago, Illinois, USA, 2022), and data were graphed using GraphPad Prism (v.9) and Adobe Illustrator 2023 (Adobe Inc., San Jose, California, USA, 2023). A two-sided *p*-value ≤0.05 was considered statistically significant.

## Results

### Animal inclusion

A total of 13 lambs (from seven ewes) were instrumented, but two lambs could not be included due to surgical complications prior to initiation of the experiments. The remaining 11 lambs were randomised to PBCC (*n* = 5) or ICC (*n* = 6) groups. Samples for PGE_2_ and PGEM concentrations were available from four PBCC lambs and six ICC lambs.

### Baseline characteristics

Baseline characteristics are displayed in Table 1. Baseline characteristics were similar between PBCC and ICC lambs. The majority of both PBCC (80%) and ICC lambs (83%) were male with similar body and lung weights between groups (Table 1). Fetal arterial blood gas parameters were similar between PBCC and ICC lambs (Table 1).

**Table 1:** Baseline characteristics of PBCC and ICC lambs.

	**PBCC (*n*** **=** **5)**	**ICC (*n*** **=** **6)**
Male	4 (80%)	5 (83%)
Body weight (kg)	4.2 ± 0.4	4.6 ± 1.0
Lung weight (g)	148 ± 37	148 ± 25
Fetal PaO_2_ (mmHg)	22 ± 4	19 ± 7
Fetal PaCO_2_ (mmHg)	66 ± 6	66 ± 6
Fetal SaO_2_ (%)	56 ± 11	47 ± 22
Fetal Hb (g/dL)	11.9 ± 1.3	12.5 ± 0.9
Fetal Hct (%)	37 ± 4	39 ± 3
Fetal pH	7.24 ± 0.07	7.26 ± 0.03

Data was presented as *n* (%) and mean ± standard error of the mean (SEM).

### Blood sampling

A reference blood sample was withdrawn prior to starting the protocol (fetal sample), followed by blood samples taken 30 ± 7 s after cord clamping and 33 ± 6 s after ventilation onset. A final, “lung aeration” sample was collected at 199 ± 25 s after ventilation onset, which was the average time it took for PBF to increase and abolish retrograde flow (lung aeration sample). Blood flow measurements are displayed in Supplementary File S2 (Figure S1).

### Fetal PGE2 and PGEM concentrations

*In utero*, PGE_2_ concentrations in the umbilical vein were significantly higher than in the fetal carotid (*p* = 0.020; Figure 1A) and pulmonary arteries (*p* = 0.013; Figure 1A) of all lambs. In the fetal sample, PGE_2_ concentrations were also higher in the carotid artery compared to the pulmonary artery in 5/6 lambs (*p* = 0.080; Figure 1A). In contrast, PGEM concentrations in the umbilical vein were similar to the concentrations measured in the carotid (656 ± 502 vs. 611 ± 401 pg/mL, *p* = 0.495; Figure 1B) and pulmonary arteries (656 ± 502 vs. 656 ± 377 pg/mL, *p* = 0.999; Figure 1B). PGEM concentrations in the fetal carotid and pulmonary artery were also similar (611 ± 401 vs. 656 ± 377, *p* = 0.678; Figure 1B).

**Figure 2:**
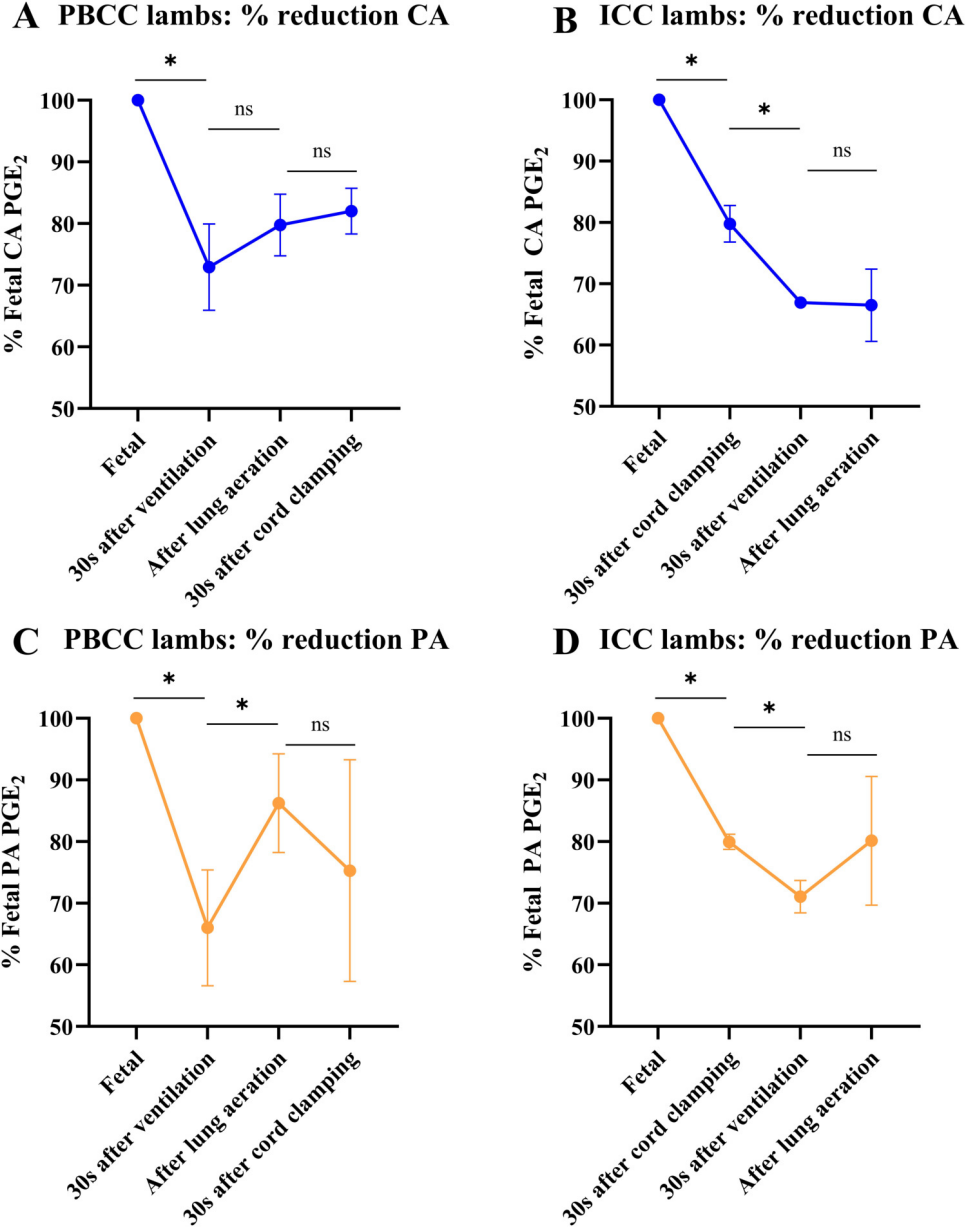
Prostaglandin (PGE_2_) changes in response to cord clamping, ventilation onset and lung aeration. PGE_2_ concentrations, expressed as a percentage of fetal concentrations, in lambs that received physiological-based cord clamping (PBCC, *n* = 4; **A** and **C**) or immediate cord clamping (ICC, *n* = 6; **B** and **D**) at birth. Concentrations were measured in both the carotid (**A** and **B**) and pulmonary (**C** and **D**) artery. Data were presented as mean ± standard error of the mean and were analysed by a One-Way Repeated Measures ANOVA followed by *post-hoc* Fisher's least square differences test between each consecutive timepoint. * represents a *p*-value ≤0.05; ns represents a *p*-value >0.05.

### Changes in PGE_2_ concentrations

PGE_2_ concentrations after birth are displayed in Supplementary File S2 (Figure S2, S3), providing absolute values for reference. For analyses, PGE_2_ concentrations relative to fetal concentrations are displayed in Figure 2. In PBCC lambs, ventilation onset significantly reduced PGE_2_ concentrations, expressed relative to fetal concentrations, in both the carotid (100 ± 0 vs. 82 ± 4%, *p* = 0.036; Figure 2A) and pulmonary (100 ± 0 vs. 66 ± 9%, *p* = 0.052; Figure 2C) arteries. While PGE_2_ concentrations in the carotid artery did not change following lung aeration and cord clamping, PGE_2_ concentrations in the pulmonary artery significantly increased once the lungs were aerated (66 ± 9 vs. 86 ± 8%, *p* = 0.012; Figure 2C).

**Figure 3:**
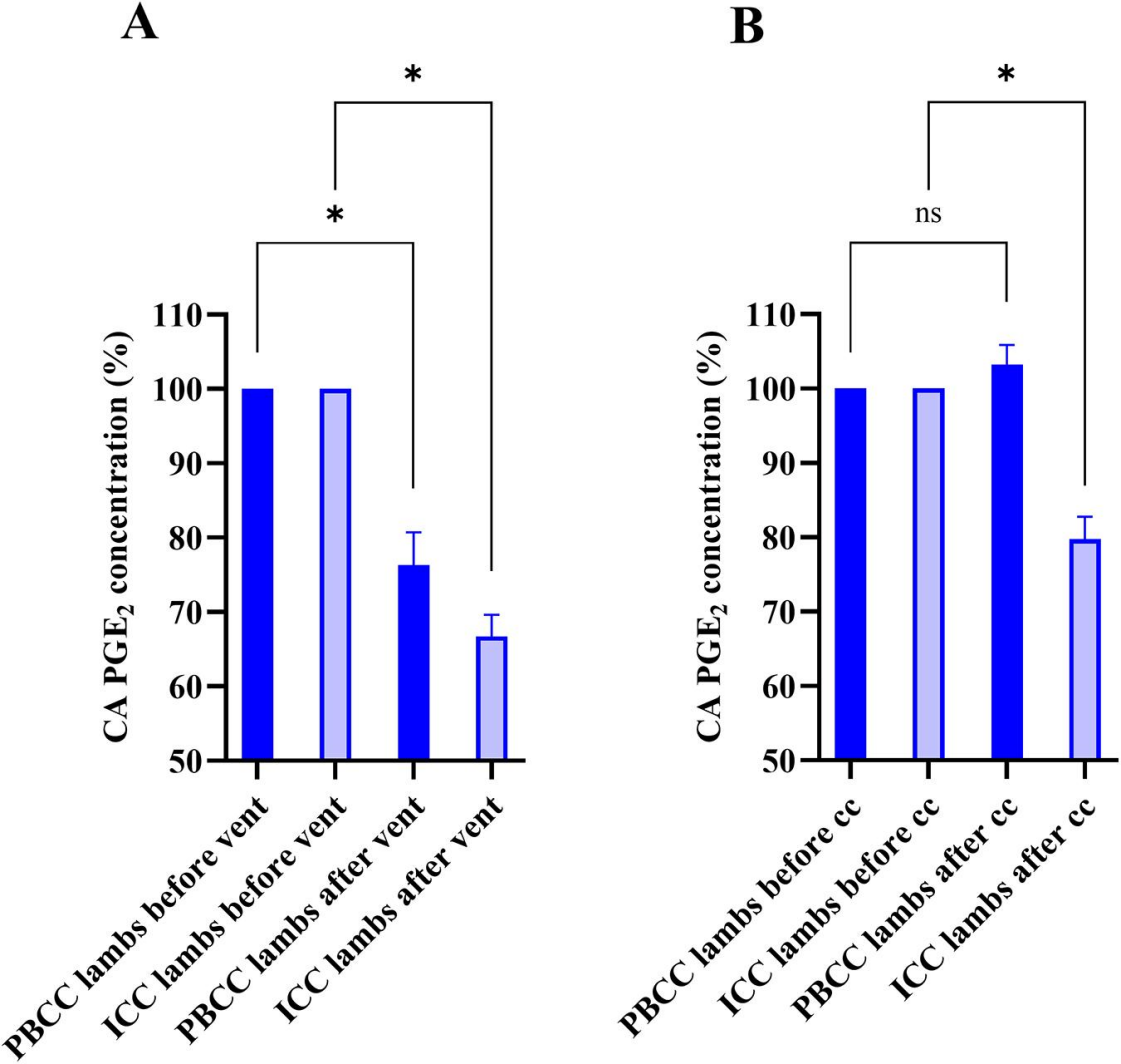
Effect of ventilation onset, lung aeration and cord clamping on prostaglandin E_2_ (PGE_2_) concentrations in the carotid artery. PGE_2_ concentrations in the carotid artery (CA) expressed as the percentage of the sample taken before **(A)** ventilation and lung aeration (vent) and **(B)** before cord clamping (cc) in both physiological-based cord clamping (PBCC, *n* = 4; orange closed bars) and immediate cord clamping (ICC, *n* = 6; orange open bars) lambs. “Before vent” samples consisted of the fetal sample in PBCC lambs or the 30 s after cord clamping sample in ICC lambs. “After vent” sample consisted of the combined mean of the ventilation onset and lung aeration sample in PBCC or ICC lambs. “Before cc” samples consisted of the lung aeration sample in PBCC lambs or the fetal sample in ICC lambs. “After cc” samples consisted of the 30 s after cord clamping sample in PBCC or ICC lambs. Data were presented as mean ± standard error of the mean. * represents a *p*-value ≤0.05; ns represents a *p*-value >0.05.

In ICC lambs, both cord clamping (100 ± 0 vs. 80 ± 3%, *p* = 0.001; Figure 2B) and ventilation onset (80 ± 3 vs. 67 ± 1%, *p* = 0.002; Figure 2B) decreased PGE_2_ concentrations, expressed relative to fetal concentrations, in the carotid artery. Similarly, PGE_2_ concentrations in the pulmonary artery also decreased after cord clamping (100 ± 0 vs. 80 ± 1%, *p* < 0.001; Figure 2D) and ventilation onset (80 ± 1 vs. 71 ± 3%, *p* = 0.014). PGE_2_ concentrations did not significantly change in the carotid or pulmonary arteries following lung aeration.

### Effect of pulmonary ventilation and cord clamping

**Figure 4:**
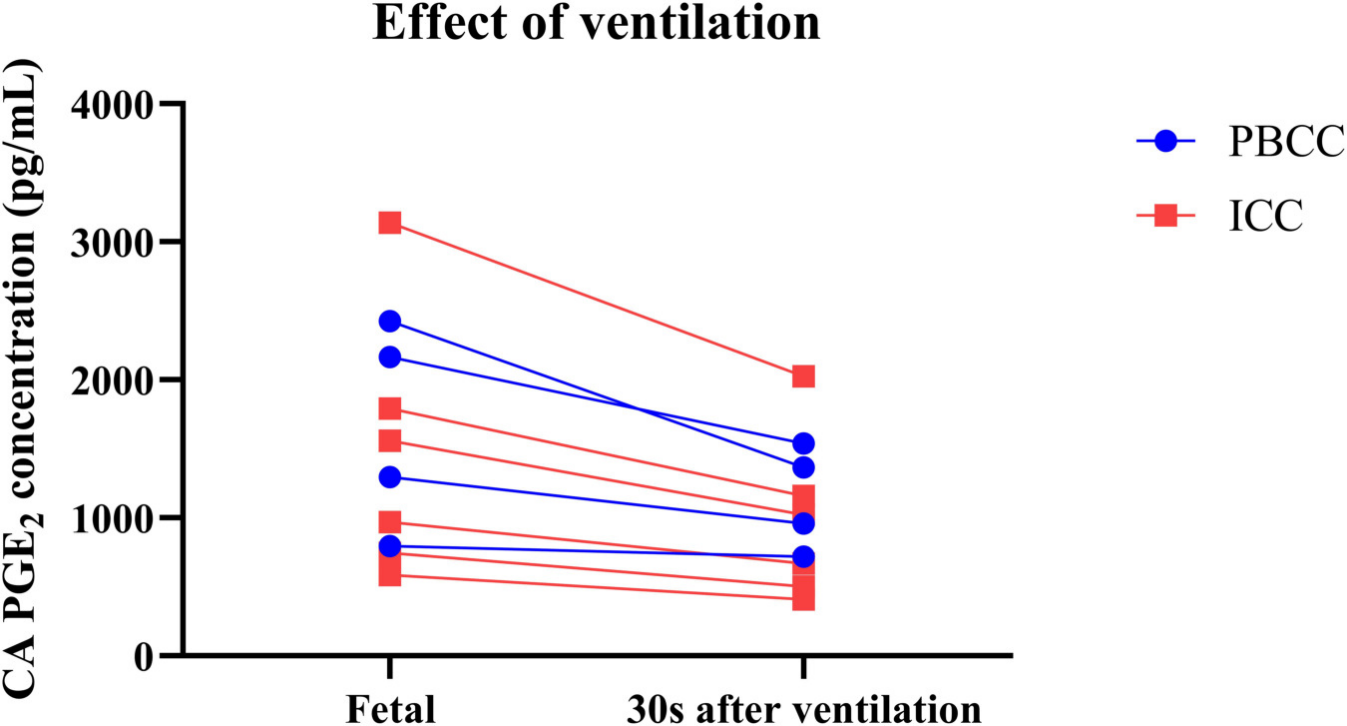
Prostaglandin E_2_ (PGE_2_) concentrations before and after ventilation onset. PGE_2_ concentrations in the carotid artery (CA) of lambs that received physiological-based cord clamping (PBCC, *n* = 4; blue) or immediate cord clamping (ICC, *n* = 6; red) immediately before and 30 s after ventilation onset.

In PBCC lambs, ventilation of the lung (combined mean of ventilation onset and lung aeration samples from the carotid artery) significantly reduced PGE_2_ concentrations by 24 ± 4% (*p* = 0.012; Figure 3A) compared to fetal concentrations. However, PGE_2_ concentrations were not decreased further in response to cord clamping (lung aeration vs. cord clamping %; 100 ± 0 vs. 103% ± 3%, *p* = 0.314; Figure 3B). The effect of ventilation on PGE_2_ concentrations in individual lambs is displayed in Figure 4.

In ICC lambs, cord clamping reduced PGE_2_ concentrations by 20% ± 3% relative to fetal concentrations (*p* = 0.001; Figure 3A) and subsequent ventilation of the lung (combined mean of ventilation onset and lung aeration samples) from the carotid artery also significantly reduced PGE_2_ concentrations by 33% ± 3% (*p* < 0.001; Figure 3B).

### Changes in PGEM concentrations

In PBCC lambs, PGEM concentrations in the carotid and pulmonary arteries, displayed in Supplementary File S2 (Figure S2), did not significantly change in response to ventilation onset (carotid artery: *p* = 0.265; pulmonary artery: *p* = 0.925), lung aeration (*p* = 0.334; *p* = 0.829), and cord clamping (*p* = 0.460; *p* = 0.233). Similarly, in ICC lambs, PGEM concentrations in the carotid and pulmonary artery did not change in response to cord clamping (*p* = 0.444; *p* = 0.771), ventilation onset (*p* = 0.362; *p* = 0.219), and lung aeration (*p* = 0.420; *p* = 0.165) [Supplemental file S2 (Figure S2)].

## Discussion

This study demonstrates that circulating prostaglandin E_2_ (PGE_2_) concentrations, but not its metabolite (PGEM), decrease during the fetal-to-neonatal transition at birth, largely in response to ventilation onset and cord clamping. To separate the independent effects of cord clamping and ventilation onset on circulating PGE_2_ concentrations, blood samples were collected following both PBCC (ventilation onset prior to cord clamping) and ICC (cord clamping prior to ventilation onset) in premature lambs. During PBCC, it was found that circulating PGE_2_ concentrations in the carotid artery decreased in response to ventilation onset and lung aeration compared to fetal concentrations, but not did not decrease further in response to cord clamping. In contrast, cord clamping prior to ventilation onset significantly decreased PGE_2_ concentrations and PGE_2_ concentrations were further reduced by ventilating and aerating the lung. Therefore, while the effects of cord clamping depend on its timing, ventilation onset and subsequent lung aeration consistently decrease PGE_2_ concentrations, which would be expected to promote spontaneous breathing after birth.

Circulating PGE_2_ concentrations are much higher in the fetus than in the neonate (16, 17, 28), likely due to high production rates and the release of PGE_2_ into the fetal circulation by the placenta. This explains the much higher PGE_2_ concentrations in the umbilical vein compared with other fetal vessels (14). Higher circulating PGE_2_ concentrations in the fetus are also thought to result from reduced prostaglandin metabolism rates. This is due to redirection of right ventricular output away from the fetal lungs (and through the ductus arteriosus), the primary site of prostaglandin metabolism in the adult (23). As a result, *in utero*, PGE_2_ is thought to act as a circulating hormone that has a variety of actions including regulating FBMs, organ maturation, thermogenesis, patency of the ductus arteriosus, glucose homeostasis, stress hormone concentrations, and may even contribute to the onset of labour (29). Circulating PGE_2_ concentrations also increase in response to fetal hypoxia (30, 31) and although it is not clear what role they play, the inhibition of prostaglandin synthesis during fetal hypoxia causes severe acidaemia and fetal demise (32). Similarly, PGE_2_ also plays an important role in the fetal response to intrauterine inflammation, particularly in the lung (33). However, relevant to this study is PGE_2_'s well-established inhibitory effect on FBMs, which could restrict the onset of continuous breathing at birth, if postnatal concentrations remained elevated (29). As the onset of continuous breathing is critical for postnatal survival, exposure of the newborn to inhibitory factors, such as hypoxia and elevated PGE_2_ concentrations, should be avoided or minimised. Based on our findings, this can simply be achieved by aerating the lungs before cord clamping, which would be expected to increase both oxygenation and decrease PGE_2_ concentrations by increasing its metabolism. While clamping the cord will also decrease PGE_2_ supply, this provides no additional benefit with regard to reducing PGE_2_ concentrations in the immediate newborn period.

In contrast to the fetus, PGE_2_ is thought to act in a more paracrine fashion in neonates and adults, largely due to the highly efficient metabolism of PGE_2_ as it passes through the lungs (16, 17, 23, 24, 34). The current study found that PGE_2_ concentrations decreased in the carotid and pulmonary arteries following cord clamping in ICC lambs, although this effect of cord clamping was masked if it was preceded by ventilation onset. By removing the placental source of PGE_2_, the decrease in PGE_2_ concentrations in ICC lambs is likely due to ongoing metabolism, as otherwise concentrations would remain stable. However, as PGE_2_ concentrations decreased immediately after cord clamping and before ventilation onset, increased metabolism by the lung could not be responsible. Instead, this most likely occurred in the liver, which is known to have high 15-PGDH levels, the enzyme largely responsible for prostaglandin metabolism (35, 36). While PGE_2_ concentrations decreased following cord clamping (and ventilation onset), concentrations rapidly stabilised which is indicative of ongoing production from sources other than the placenta. However, following ventilation onset in PBCC lambs, we were unable to detect a decrease in PGE_2_ concentrations in response to cord clamping. The disparity was presumably due to a combination of factors associated with ventilation onset. These include a reduction in umbilical venous flow associated with the onset of left-to-right ductal shunting and the redirection of left and right ventricular output through the lungs, rather than through the placenta, following lung aeration (37, 38). In addition, the increase in pulmonary venous return to the left atrium, which has lower PGE_2_ concentrations, likely overwhelms the high PGE_2_ influx of the (already reduced) umbilical venous blood flow entering via the foramen ovale. As a result, the impact of cord clamping on PGE_2_ concentrations was not detectable.

By aerating the lung, ventilation onset greatly increases PGE_2_ metabolism at birth by stimulating a large decrease in pulmonary vascular resistance, which redirects 100% of right ventricular output through the lungs. In addition, the onset of left-to right shunting through the ductus arteriosus means that a large proportion of left ventricular output will also pass through the lungs (37). As the lung is the primary site of prostaglandin metabolism in the adult/newborn (23), the huge (30-fold) increase in blood passing through the lungs explains why a rapid decrease in PGE_2_ concentrations following ventilation onset was observed. This result is consistent with our finding of marked 15-PGDH staining in lung tissue from these preterm newborn lambs, which is primarily found in distal, rather than proximal, airways (22). Furthermore, a reduction in umbilical venous flow may also contribute to less influx of PGE_2_ into the circulation of PBCC lambs following ventilation onset. It is interesting that, in ICC lambs, cord clamping decreased PGE_2_ concentrations, which were further decreased by ventilation onset. This result clearly demonstrates that increased PGE_2_ metabolism via the lung is a major contributor to the decrease in circulating PGE_2_ concentrations after birth, whereas the effect of cord clamping is complicated by the presence or absence of lung aeration.

Our findings confirm and extend those of other experimental studies that reported a decrease in PGE_2_ concentrations after birth (16, 17, 24); though concentrations were much higher in the current study and more reflective of fetal concentrations. The concentration differences are likely due to different sampling positions and sampling time points. Indeed, we measured PGE_2_ concentrations both before and in the first few minutes after birth, whereas other experimental studies have evaluated concentrations hours-to-days after birth, when we would expect that PGE_2_ clearance would be much greater (16, 17, 24). In this experiment, we chose to compare changes in PGE_2_ concentrations relative to fetal levels measured in the same animal, to counter the variability in PGE_2_ concentrations between animals [Supplementary File S2 (Figure S3)]. As circulating fetal PGE_2_ concentrations are known to depend on factors such as time of day, nutritional status and oxygenation level, large variations in circulating concentrations between animals are expected (14, 32). However, despite this large variability in concentrations between animals, the changes in PGE_2_ concentrations in response to cord clamping and ventilation onset within each animal were remarkably similar (Figure 4). Indeed, all ICC lambs reacted similarly to cord clamping and ventilation onset (Figure 4), although this variability tended to increase following lung aeration. This likely reflects the variability in the degree of lung aeration and increase in PBF between animals, which also likely explains the slightly higher variability in percentage change observed in PBCC lambs (Figures 1A,C) than ICC lambs.

In premature infants, delayed cord clamping strategies (including PBCC) can significantly reduce mortality and the need for blood transfusions compared to earlier cord clamping strategies (39, 40). As PGE_2_ is an inflammatory mediator known to inhibit respiratory drive (11, 41, 42) and PGE_2_ concentrations decrease with ventilation onset (current study), this highlights the importance of stimulating and supporting spontaneous breathing at birth when cord clamping is delayed. By stimulating and supporting spontaneous breathing, clinicians can assist in establishing lung aeration and commence a positive feedback mechanism with subsequent decreases in PGE_2_ concentration and promotion of spontaneous breathing (21).

As the placenta is the primary source of circulating PGE_2_ in the fetus, it is logical that PGE_2_ concentrations were higher in the carotid than pulmonary artery in 5/6 lambs. This is because umbilical venous return mostly passes through the ductus venosus and foramen ovale to directly enter the left atrium (43). As a result, like oxygen levels, PGE_2_ concentrations in the carotid artery should be higher than in the pulmonary artery. Interestingly, the significant increase in PGE_2_ concentrations in the pulmonary artery between ventilation onset and lung aeration in the PBCC lambs was probably due to the onset of left-to-right shunting through the ductus arteriosus. As the samples were collected from a catheter with its tip in the left pulmonary artery, distal to its junction with the ductus arteriosus, the large contribution of left ventricular output to PBF would be expected to increase PGE_2_ concentrations to similar levels as the carotid artery. It is also possible that the redirection of umbilical venous return into the right atrium, rather than through the foramen ovale, may also contribute to higher PGE_2_ concentrations in the pulmonary artery following ventilation onset (44). While overall fetal PGE_2_ metabolism must equal placental PGE_2_ production, otherwise concentrations would continue to increase, the difference in PGE_2_ concentrations in the umbilical vein and fetal arteries is likely due to dilution as it enters the fetal circulation, but could in part also be explained by hepatic metabolism, as discussed above.

It is interesting that PGEM concentrations remained relatively stable throughout the sampling protocol in both PBCC and ICC lambs, particularly as an increase in metabolism partly explains the decrease in PGE_2_ concentrations at birth. However, PGEM has a much longer half-life than PGE_2_ so PGEM concentrations are unlikely to change as rapidly as PGE_2_ concentrations (45). It is also unclear how quickly metabolised PGE_2_ re-enters the circulation as PGEM and if it takes more than a few minutes, then it is not surprising that we did not detect the contribution of metabolised PGE_2_ to circulating PGEM concentrations following ventilation onset.

This study is mostly limited by the small sample size as well as missing data that lower our statistical power. PGE_2_ concentrations in this study were elevated compared to clinical and experimental studies (16–18, 24, 28, 46), but given the consistency of PGE_2_ changes between animals, we consider that the relative changes accurately reflect the changes in PGE_2_ concentrations directly at birth.

In summary, we have shown that PGE_2_ concentrations change within 30 s in the carotid and pulmonary arteries of premature lambs in response to umbilical cord clamping and ventilation onset at birth, whereas PGEM concentrations do not. Ventilation onset and lung aeration likely reduce PGE_2_ concentrations by increasing PBF and thereby increasing PGE_2_ metabolism within the lung. Decreases in PGE_2_ concentrations associated with cord clamping are most likely due to endogenous metabolism following cessation of the supply of high PGE_2_ concentrations from the placenta. While ventilation onset reduced PGE_2_ concentrations irrespective of cord clamping timing, ventilation onset and subsequent lung aeration tended to obscure the effect of cord clamping on circulating PGE_2_ concentrations, likely due to the circulatory changes induced by ventilation onset.

## Data Availability

The original contributions presented in the study are included in the article/Supplementary Material, further inquiries can be directed to the corresponding author.
