# Supplementary material for: Circulating prostaglandin E2 concentrations decrease at birth in premature lambs
Source: Front Pediatr. 2025 Nov 28;13:1636459. doi: 10.3389/fped.2025.1636459 (PMC12698598; doi:10.3389/fped.2025.1636459)
Supplement: Supplementary file 1 [file Datasheet1.docx]

## PGE_2_ concentration analysis

The blood samples were analysed to determine the primary outcome of transitional PGE_2_ concentrations (pg/mL) using a commercially available monoclonal PGE_2_ ELISA kit (cat# 514010, Cayman Chemicals, Ann Arbor, MI, United States) according to the manufacturer’s instructions. In brief, after sampling, indomethacin was added to the blood samples to give a final concentration of at least 10 μM to prevent ex vivo formation of prostaglandins. The blood samples were then centrifuged, plasma collected and stored at -80 °C. Fifty μL of sample were assayed in triplicate with tracer and monoclonal antibodies. The plate was incubated in the dark for 18 hours at 4 °C. All plates were washed to remove unbound reagents. Ellman’s reagent was added to each well and incubated for 1-1.5 hours at room temperature in the dark. Absorbance was measured at 412 nm using a plate reader (SpectraMax i3, Molecular Devices, San Jose, CA, United States). Internal quality control was maintained throughout the assay, ensuring that all measured values remained within the detection limits. The intra-assay and inter-assay coefficient for variability was maintained at <20%. Assay range was 7.8-1000 pg/mL with a sensitivity (80% B/B_0_) of approximately 13 pg/mL.

## PGE metabolite (PGEM) concentration analysis

PGEM concentrations were determined via a commercially available monoclonal ELISA kit (cat# 514531, Cayman Chemicals, Ann Arbor, MI, United States) according to manufacturer’s instructions. Fifty μL of sample were assayed in triplicate with a tracer and monoclonal antibodies. Each plate was incubated for 18 hours at room temperature. All plates were washed to remove unbound reagents and Ellman’s reagent was added to each well and incubated at room temperature for 1.5 hours. Plates were read at 410 nm on a plate reader (SpectraMax i3, Molecular Devices, San Jose, CA, United States). Internal quality controls were included with all PGEM concentrations measured within the kit’s detection range. Intra-assay and inter-assay coefficient of variability was set <20%, assay range was set from 0.39-0.50 pg/mL and average sensitivity (defined as 80% B/B_0_) was 2 pg/mL.

## 15-hydroxide prostaglandin dehydrogenase (15-PGDH) detection

At postmortem examination, the lungs were dissected from the chest and the right lung fixed by airway instillation with 10% formalin at 20cmH_2_O. After fixation, the lung was separated into upper, middle and lower lobes, sectioned into 1.5cm slices with random sections from each lobe taken and post fixed in Zambonis fixative, which were then processed and mounted into paraffin blocks. After histological processing, blocks were randomly selected for immunohistochemical detection of PDGH.

4 μm lung sections were cut using a rotary microtome (Reichert-Jung Biocut 2030), Lecia, Germany) and mounted onto glass slides (Superfrost Plus, Thermo Scientific, Germany) and heated in a tissue-drying oven at 60 °C for 45 minutes. Slides were dewaxed in three changes of xylene, and rehydrated with decreasing concentrations of ethanol.

To retrieve antigens, slides were heated to 99-100°C in 0.01 M sodium citrate buffer (pH 6.0 for 20 minutes. Sections were washed with TBS with Tween (TBST), incubated in hydrogen peroxide (3% x 20min), washed in TBST (3 x 5 mins) and incubated in universal blocking buffer (DAKO cat#X0909) for 30 minutes. All sections were incubated with primary antibody (1:100 Rabbit Polyclonal Anti-15-PGDH, cat #LS-B6957, LifeSpan Bioscience) in a humified chamber at 4°C overnight. Sections were rinsed with TBST (3 x 1 min) and incubated with secondary antibody (1:700 biotinylated Goat Anti-rabbit IgG , cat #BA-1000,VectorLabs) for 45min at room temperature before being incubated with . the avidin-biotin complex (1:150, Sigma Aldrich) for 45 min at room temperature. Sections were washed in TBST (3 x 1 min). Sections were reacted with 3,3’-diaminobenzidine tetrahydrochloride (Sigma Aldrich) for 3 min. Sections were counterstained with haematoxylin (Amber Scientific), dehydrated and cover-slipped. Negative control slides confirmed the specificity of immunohistochemical staining.
