# Supplementary material for: Circulating prostaglandin E2 concentrations decrease at birth in premature lambs
Source: Front Pediatr. 2025 Nov 28;13:1636459. doi: 10.3389/fped.2025.1636459 (PMC12698598; doi:10.3389/fped.2025.1636459)
Supplement: Supplementary file 2 [file Datasheet2.docx]

Supplemental Figure 1: Carotid artery and pulmonary artery blood flow changes during the fetal-to-neonatal transition

Carotid artery blood flow (CBF; blue) and pulmonary artery blood flow (PBF; orange) in lambs that received physiological-based cord clamping (PBCC, n=5; A) or immediate cord clamping (ICC, n=6; B) during the transition at birth. Lung aeration was defined by a large increase in PBF, with an absence of retrograde flow (i.e. blood flow out of the lungs) during diastole. Data were presented as mean ± standard error of the mean.

Supplemental Figure 2: Changes in Prostaglandin E_2_ (PGE_2_) and Prostaglandin E metabolite (PGEM) concentrations during the fetal-neonatal transition

PGE_2_ (blue and orange) and PGEM (green and purple) concentrations in lambs that received physiological-based cord clamping (PBCC, n=4; A and C) or immediate cord clamping (ICC, n=6; B and D). Concentrations were measured in both the carotid artery (A and B) and pulmonary artery (C and D). Data were presented as mean ± standard error of the mean.

Supplemental Figure 3: Immunohistochemical staining for the enzyme 15-hydroxyprostaglandin dehydrogenase (15-PGDH)


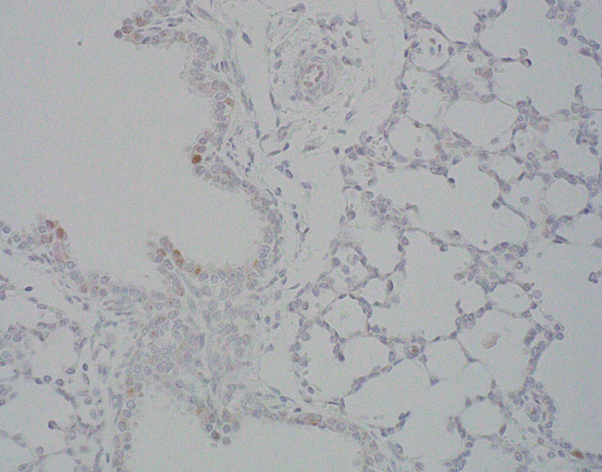


Representative image of 15-PGDH stained lung section of premature lambs.
